# Bacterial Cellulose (*Komagataeibacter rhaeticus*) Biocomposites and Their Cytocompatibility

**DOI:** 10.3390/ma13204558

**Published:** 2020-10-14

**Authors:** Valentina A. Petrova, Albert K. Khripunov, Alexey S. Golovkin, Alexander I. Mishanin, Iosif V. Gofman, Dmitry P. Romanov, Alexandra V. Migunova, Natalia A. Arkharova, Vera V. Klechkovskaya, Yury A. Skorik

**Affiliations:** 1 Institute of Macromolecular Compounds of the Russian Academy of Sciences, Bolshoy ave. V.O. 31, 199004 St. Petersburg, Russia; valentina_petrova_49@mail.ru (V.A.P.); biocell@imc.macro.ru (A.K.K.); gofman@imc.macro.ru (I.V.G.); 2 Almazov National Medical Research Centre, Akkuratova str. 2, 197341 St. Petersburg, Russian; golovkin_a@mail.ru (A.S.G.); mishaninssma@yandex.ru (A.I.M.); 3 Institute of Silicate Chemistry of the Russian Academy of Sciences, Adm. Makarova emb. 2, 199034 St. Petersburg, Russia; dprom@mail.ru; 4 Department of Microbiology, Faculty of Biology, St. Petersburg State University, Universitetskaya emb. 7-9, 199034 St. Petersburg, Russia; sasha_mig_2405@mail.ru; 5 Shubnikov Institute of Crystallography, Federal Scientific Research Centre “Crystallography and Photonics”, Russian Academy of Sciences, Leninskiy ave. 59, 119333 Moscow, Russia; natalya.arkharova@yandex.ru (N.A.A.); klechvv@crys.ras.ru (V.V.K.)

**Keywords:** bacterial cellulose, *Komagataeibacter rhaeticus*, chitosan, hyaluronan, alginate, carrageenan, biocomposites, biocompatibility

## Abstract

A series of novel polysaccharide-based biocomposites was obtained by impregnation of bacterial cellulose produced by *Komagataeibacter rhaeticus* (BC) with the solutions of negatively charged polysaccharides—hyaluronan (HA), sodium alginate (ALG), or κ-carrageenan (CAR)—and subsequently with positively charged chitosan (CS). The penetration of the polysaccharide solutions into the BC network and their interaction to form a polyelectrolyte complex changed the architecture of the BC network. The structure, morphology, and properties of the biocomposites depended on the type of impregnated anionic polysaccharides, and those polysaccharides in turn determined the nature of the interaction with CS. The porosity and swelling of the composites increased in the order: BC–ALG–CS > BC–HA–CS > BC–CAR–CS. The composites show higher biocompatibility with mesenchymal stem cells than the original BC sample, with the BC–ALG–CS composite showing the best characteristics.

## 1. Introduction

Bacterial cellulose (BC) is a linear, non-branched polysaccharide consisting of β-1,4-glucopyranose units and is produced extracellularly by certain microorganisms through oxidative fermentation. *Komagataeibacter rhaeticus* (also known as *Acetobacter xylinum* and *Gluconacetobacter xylinus*) is the best studied and the most efficient BC producer, as it has the capacity to assimilate several different sugars and yields high levels of cellulose in liquid culture medium [1]. However, the ability to synthesize cellulose as a component of the extracellular capsular polysaccharide is also known in the genera *Pseudomonas*, *Achromobacter*, *Alcaligenes, Azotobacter*, *Zooglea*, and *Sarcina ventriculi* [1], as well as, under certain conditions, *Salmonella typhimurium* and *Escherichia coli* [2].

BC has highly unique characteristics, such as strong water retention, slow water liberation, high crystallinity, excellent thermal and mechanical properties, an ultra-dispersed fiber network, multifunctionality, absence of toxicity, and the ability to mold into 3D structures [3]. These features make it a promising material for use in biomedicine as drug carrier systems, matrices for tissue engineering, wound dressing materials, vessel implants, artificial blood vessels, biological films, and biosensors [4,5,6,7,8,9,10]. BC is highly biocompatible due to its structural similarity to components of the extracellular matrix (e.g., collagen), and it undergoes complex interactions with biological tissues. The application of BC as scaffolds for tissue regeneration has been investigated for bone tissue, cartilage, muscles, skin tissue, and dental applications [11,12,13,14]. The influence of BC morphology on cell proliferation has been estimated in a number of research studies [1,15]; however, the limiting factor for the biomedical use of BC continues to be its low biodegradability [16,17]. Recently, Ivanova et al. [18] used enzymatic hydrolysis with a cellobiohydrolase from *Scytalidium candidum 3C* to change the crystallinity, supramolecular structure, and properties of BC. Their results suggest the possibility of developing biodegradable BC-based dressings for biomedical purposes.

BC can be fabricated into a diversity of composite materials by varying the method of introduction of companion biopolymers and their fixation into the BC structure (e.g., polymer adsorption, wet molding of BC dispersed in polymer solutions, biogenesis of BC in the presence of polymers, or combinations of these methods). The porous geometry of BC and its ability to adsorb different materials make BC a promising component for the production of composites that may show better properties and additional functionalities compared to BC on its own. A recent review [19] discusses the influence of BC modification on its morphology and confirms that nanosized BC composites could be obtained by manipulating BC biogenesis (i.e., simultaneous crystallization of BC and a polymer during the formation of a ribbon). BC morphology can be further changed by altering the conditions of its cultivation, as a high oxygen content during static cultivation favors an increase in BC density and, correspondingly, in the mechanical strength of the resulting BC scaffold [20,21].

BC-based composite materials with different morphologies and properties have been formulated using the following biologically active polysaccharides: chitosan (CS) [22,23], sodium alginate (ALG) [24,25], sodium hyaluronate (HA) [26], carrageenan (CAR) [25], gellan gum [25], guar gum [26], cellulose derivatives [27], and potato starch [28]. For example, a BC–CS composite was prepared by immersion of a wet BC film into a CS solution, followed by freeze-drying [22]. SEM images of the composite surface showed that CS penetrates into the BC to form a three-dimensional multilayer network. The resulting scaffold had a very well interconnected porous structure and a large aspect surface. When CS is incorporated into BC, crystallinity tends to decrease and thermal stability increases. Cytocompatibility with fibroblast cells (3 T3) was much better for the BC–CS scaffold than for pure BC.

BC composites have also been prepared with hydrocolloids (guar gum and HA) and coated with collagen or not containing collagen [26]. The composite scaffolds showed good stability in physiological saline and had better swelling than the original BC. The adhesion and proliferation of L929 mouse fibroblasts onto the surfaces of BC composites was equivalent to BC but had the advantage of more uniform cell distribution, and the cell morphology indicated a favorable environment for the development of fibroblasts. A relationship was found between the surface energy and cell viability, as well as between the surface energy and the content of hydrocolloids. Therefore, these properties can also be controlled by changing the content of hydrocolloids in the composite.

A previous study [24] evaluated various porous bacterial nanocellulose scaffolds for 3D adipose tissue. The 3D scaffolds were engineered by crosslinking homogenized cellulose fibrils using alginate and then freeze drying the mixture to obtain a porous structure. Varying the ratio between BC and ALG and using various freezing techniques led to significant differences in the properties of the composites. The smaller pore size was more attractive to the cells and led to a denser cell population. The authors [24] concluded that 3D culturing of adipocytes in BC scaffolds is a promising method for the fabrication of adipose tissue that can serve as an in vitro model for adipose biology and metabolic disease. Thus, previous studies have shown that BC–polysaccharide composite scaffolds are biologically active and suitable for cell adhesion; therefore, they have potential for use in regenerative medicine and tissue engineering.

The aim of the present work was to investigate BC-based biocomposites that contain polyelectrolyte complexes (PEC) sandwiched within anionic polysaccharides (HA, ALG, and CAR) and a cationic polysaccharide (CS). The structure, morphology, and physicochemical characteristics of the obtained composites were evaluated, as was their compatibility with cultured multipotent mesenchymal stem cells (MMSCs). The composites examined here consist of polysaccharides carrying opposite charges, and they form a polyelectrolyte network without requiring the use of potentially dangerous crosslinking agents. The presence of PEC between biologically active polysaccharides may also change the BC network architecture and impart new and useful properties to cellulose.

## 2. Materials and Methods

### 2.1. Polysaccharides

The biosynthesis of BC was carried out under surface cultivation using a B-13015 strain of *Komagataeibacter rhaeticus* (Russian National Collection of Industrial Microorganisms), according to the previously published method [29]. Briefly, a gel film of the required thickness (7 mm) was formed by surface cultivation of bacteria on Scar–Hestrin nutrient medium. The composition of the medium was 2.0% glucose, 0.5% yeast extract, 0.5% peptone, 0.15% citric acid, and 0.27% Na_2_HPO_4_. Cultivation of the bacteria was stopped upon reaching the required gel film thickness. The gel film was then washed with distilled water to remove residual culture medium and boiled in 0.5% NaOH to remove non-cellulosic components, followed by thorough washing in distilled water. The thus obtained BC gel film was then squeezed between layers of cotton fabric on an oil press until threefold weight loss (henceforth, wrung-out BC).

The BC-containing composites were prepared using the following polysaccharides: chitosan (Ennagram, Pantin, France) with a molecular weight (MW) of 1.6 × 10^5^ and a degree of deacetylation of 80% [30]; sodium hyaluronate (Shandong Focuchem Biotech Co., Jining, China) with a MW of 5.4 × 10^4^ [30]; carrageenan with a MW = 5.0 × 10^5^ (Sigma, Søborg, Denmark); and sodium alginate with a MW = 1.3 × 10^5^ (Qingdao Bright Moon Seaweed Group Co. Ltd., Qingdao, China).

### 2.2. Preparation of BC-Based Composites

The following solutions were used: 0.5% and 3% aqueous HA solutions, 2% aqueous ALG solution, 0.5% and 1.0% aqueous CAR solutions, and 1% CS in 1% acetic acid. The BC composites were prepared at room temperature (20 ± 2 °C). A gel film of wrung-out BC containing 10% dry matter was used in the preparation of composites. The composites were obtained by successive impregnation of BC with polysaccharide solutions of different concentrations; the treatment time was also varied. The wrung-out BC was placed into a solution of a given polysaccharide and exposed for a certain time. The excess solution was then removed and the sample was placed in a solution of an oppositely charged polysaccharide. As the last stage, the samples were exposed to 2% ammonia in ethanol to transform the CS into the basic form (insoluble in water). The samples were then washed with ethanol and dried at room temperature. Control experiments involved exposing the wrung-out BC to ethanol and drying at room temperature (henceforth, original BC).

For some experiments, the samples were freeze-dried using a Freeze Dryer 10N (Freeze Dryer 10N (Shanghai Drawell Scientific Instrument Co., Shanghai, China). These samples are labeled with a superscript ^FD^.

### 2.3. Characterization of Composites

The swelling degrees of the composites in water were determined gravimetrically after exposure for 24 h. The polysaccharide content in the composites was also determined gravimetrically at every stage of the treatment. X-ray diffraction analysis was performed using a DRON-3M instrument (CuK_α_ irradiation, λ = 1.5418 Å). The position of the reflections in the diffraction patterns was controlled by an external standard (quartz).

The surface morphology of the dried BC composites and their cleavages were studied by low-voltage scanning electron microscopy (SEM) with a Scios (FEI, Hillsboro, OR, USA) instrument at an accelerating voltage of 1 kV in the secondary electron mode. No coating was used.

The porosity of the samples was studied by porosimetry using a Porotech 3.1 instrument and Porovoz software, as described previously [31]. Octane was used as a wetting liquid.

Mechanical tests of the gel-films were performed in the uniaxial extension mode using an AG-100kNX Plus universal mechanical test system (Shimadzu, Kyoto, Japan). Experiments were conducted at room temperature (20 ± 2 °C). Dog-bone test samples 4 mm × 20 mm in size (gauge width and length) were stretched at a rate of 10 mm/min, according to ASTM D638 requirements. The modulus values E, the break stress σ_b_, and the ultimate deformation ε_b_ were determined.

### 2.4. Cell Cultures and In Vitro Tests

The cytocompatibility of the fabricated scaffolds was studied in cultured cells using reference samples of the original BC and cover glasses 12 mm in diameter. The cell culture consisted of human MMSCs obtained from the subcutaneous fat of healthy donors. All in vitro biological tests were performed according to the Declaration of Helsinki, and approval was obtained from the Ethics Committee of the Almazov National Medical Research Centre (No. 12.26/2014; 1 December 2014). Cells were cultivated in Minimum Essential Medium α containing fetal bovine serum (10%), 1% L-glutamine, and 1% penicillin/streptomycin and incubated at 37 °C in 5% CO_2_.

Rectangular samples with dimensions of 12 mm × 8 mm (matching the well size) and cover glasses were treated with 70% ethanol for 10 min, washed three times with phosphate buffered saline (PBS), and placed into the holes of a 24-well plate. PBS was added to the samples, and they were left to stand for 24 h. The samples and glasses were then washed once with PBS, 2 mL of cell culture medium was added to each well, and the tray was cultured for 24 h in the CO_2_ incubator at 37 °C. After 2 days, the medium was removed, 2 mL of MMSC suspension (cell concentration: 5 × 10^4^ cells/mL) was added to each well and the cells were co-cultivated for an additional 48 h. The samples and glasses were transferred into wells of a new plate, fresh cell culture medium was added, and the cells were cultured for a further 24 h. Each experiment was performed in triplicate.

After the 72 h cultivation, the samples and glasses were transferred to the wells of a new plate. Samples with adhered cells were washed off with PBS and fixed with 4% paraformaldehyde (PFA) for 10 min.

After fixation, the samples and glasses were washed with PBS to remove PFA and were treated with 0.05% Triton X-100 for 3 min, washed three times with PBS, and then treated with rhodamine-labeled phalloidin (at a dilution of 1:500 in 1% PBS). The samples were incubated for 20 min at room temperature and then washed five times with PBS. After that samples were treated with 4,6-diamidino-2-phenylindole (DAPI; dilution 1:40,000), incubated for 40 s to stain the nuclei, and then washed thoroughly with PBS to remove unreacted dye.

After staining, the samples were stored in PBS in the dark at +4 °C. Cover glasses with cells from reference wells were mounted onto microscope slides using a suitable mounting medium and stored in the dark at room temperature.

The stained MMSCs were studied by fluorescence microscopy for qualitative analysis of adhered cells. The cells were visualized with an Axiovert inverted fluorescence microscope (Zeiss, Oberkochen, Germany) equipped with a Canon camera by placing pieces of the materials bearing cells between two microscope slides. DAPI and phalloidin fluorescence were registered using the relevant filters.

Images of each sample were captured from 10 different fields of view at 10× and 40× magnification and used for qualitative analysis (cell morphology and colony formation were estimated from the stained cytoskeleton). Quantitative analysis (calculation of the number of cell nuclei stained with DAPI) was not performed due to the presence of spheroidal colonies on the surface of a majority of the samples.

The obtained data were statistically analyzed using the GraphPad Prism and Statistica 7.0 software and the Mann–Whitney non-parameter U-criterion. The results are presented as means and standard deviations (SD).

## 3. Results

### 3.1. Preparation and Swelling Properties of Composites

Water swelling is an important characteristic of materials under consideration for applications in tissue engineering, as this swelling directly relates to the transport properties of nutrients, gases, and regulatory factors and facilitates cell–biomaterial interactions and cell differentiation and proliferation. For example, moisture under a wound dressing is a desired characteristic since it accelerates epithelialization and wound healing by maintaining the proteins and cytokines produced in response to injury and facilitates autolytic debridement of the wound [26].

The hydrophilicity of BC largely depends on the method used for drying the sample. The wrung-out gel film (which contains 10% dry matter) has a swelling degree of 20 g/g in water (wrung-out BC); after freeze-drying (henceforth, the BC^FD^ sample), this parameter decreases to 1.2 g/g. The preparation of BC-containing composites involves treatment with ethanol and drying at room temperature; therefore, the wrung-out BC gel film was treated in the same way. This decreased the swelling degree to 1.9 g/g (the original BC sample). The BC composites with polysaccharides were prepared on the basis of the wrung-out BC because of the high sorption capability of BC. The properties of the obtained composites are presented in Table 1. Successive immersions of the wrung-out BC ribbon into the solution of anionic polysaccharide and then into the CS solution allowed the negatively charged groups of the polyanion to interact with the positively charged protonated amino groups of CS. As previously established [32], the strength of these interactions is determined by the amount and nature of the anionic functional groups (-COO^−^ or -OSO_3_^−^) and by the macromolecular structure of anionic polysaccharide. This strength decreases in the order of: CAR > HA > ALG [32].

Impregnation of BC with a 1% solution of CAR, followed by treatment with CS (Table 1, BC–CAR–CS*), led to the formation of a composite with a swelling degree of 1.5 g/g (in water). This value is close to the swelling degree observed for the original BC sample. This may be a result of: (i) the high viscosity of the CAR solution (which would obstruct the channels in BC and decrease the swelling degree in water); and (ii) the formation of a denser polyelectrolyte network (due to interactions between the protonated amino groups of CS and the sulfate groups of CAR). The use of 0.5% CAR for impregnation leads to a negligible increase in the swelling degree of the composite in water (Table 1, BC–CAR–CS). This, in turn, indicates that the strength of PEC between CAR and CS has a predominant influence on the swelling degree of the composite.

The composite obtained by exposure of the wrung-out BC to 3% aqueous HA, followed by treatment with CS solution (Table 1, BC–HA–CS*), demonstrates a swelling degree in water (up to 16 g/g) that is eight-fold higher than that of the original BC subjected to similar treatment. Notably, the composite samples also hold their shape and do not laminate, in contrast to BC, which laminates during swelling in water. Furthermore, the formation of the loose polyelectrolyte network (due to interactions between the positively charged amino groups of CS and the negatively charged groups of polyanions) reinforces the scaffolds. The polysaccharides that penetrate into the inter-fiber space of the BC also prevent the formation of strong hydrogen bonds during drying, thereby contributing to better swelling of the composite.

Decreasing the concentration of HA (Table 1, BC–HA–CS**) leads to a sharp decrease in the swelling degree of the composite in water. A shorter time of treatment with CS solution (Table 1, BC–HA–CS), with all other conditions being equal, causes an insignificant decrease in the swelling degree of the composite in water. Use of a 2% aqueous solution of ALG (Table 1, BC–ALG–CS) for preparing the composites yields a sample with a higher swelling degree in water (24 g/g) than can be obtained using HA; this swelling degree far exceeds that of BC. This change in swelling degree may reflect the formation of a looser polyelectrolyte network between ALG and CS, in agreement with earlier data obtained for the preparation of multilayer films of these polysaccharides [32]. Thus, the hydrophilic properties of the composites prepared by successive impregnation of BC with an anionic polysaccharide and CS are mainly controlled by the nature of the polyanion used. The swelling degrees of the composites decrease in the following order: BC–ALG–CS > BC–HA–CS > BC–CAR–CS. The data for the composition of the obtained scaffolds (Table 2) show that they are rich in anionic polysaccharides; thus, CS may possibly serve as a fixation component. Notably, the obtained composites hold their shape upon swelling; this is an advantage for uses in cell immobilization.

### 3.2. Porosity of the Composites

Estimation of the porosity of the composite materials also indicates that the composites have different structures (Table 3). The porosity depends on the type of anionic polysaccharide used in the preparation of composite material and on the type of cooperative interaction with CS. The ALG-containing composite materials possess the highest porosity (due to the presence of small pores), while the CAR-containing scaffolds show the lowest porosity. Thus, the formation of a PEC during the fabrication of composite materials “reinforces” BC and prevents the formation of strong hydrogen bonds during drying. The nature of the functional groups also has some effect on the composite structure; the domains with loose polyelectrolyte networks (e.g., the ALG-containing scaffolds) have a high swelling degree and porosity.

The original BC sample, after treatment with ethanol and drying at room temperature, showed strong intermolecular interactions. For this reason, the method is not appropriate for porosity measurements because the samples show poor wetting with the organic solvent (octane).

### 3.3. Mechanical Properties

The stiffness of the gel-film materials studied here cannot be characterized with the usual procedure of the Young’s modulus calculation as the initial slope of the stress–strain curve. The reason for this is the pronounced nonlinearity of the stress–deformation dependence. The dσ/dε value, where σ stands for stress and ε for deformation, demonstrates the progressive rise in a broad range of deformations. The modulus calculation using the Mooney–Rivlin equation gives similar results. For this reason, we selected two regions of the stress–strain curves to characterize the materials’ stiffnesses, namely from 10% to 15% and from 25% to 30% deformation. The mean slope values of the stress–strain curves at these two regions were used as the measure of the materials’ elasticity (E_10–15%_ and E_25–30%_, respectively). This approach to the characterization of the mechanical behavior of swollen polymer systems is developed in [33].

**Figure 1:**
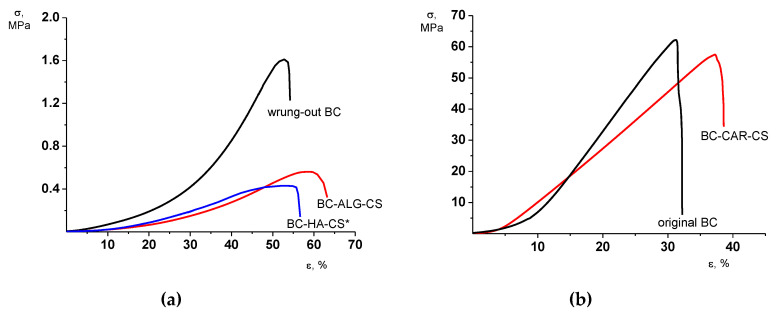
Stress–strain curves of gel-films studied: (**a**) wrung-out BC, BC–ALG–CS, and BC–HA–CS*; and (**b**) original BC and BC–CAR–CS.

All BC-based gel-films under study are characterized by a similar (at a qualitative level) character of the deformational process. A progressive increase in each material’s stiffness dσ/dε is registered along with the increase in the deformation up to the sample failure. This behavior is inherent predominantly for the films of the original BC and composite BC–HA–CS* and BC–ALG–CS (Figure 1).

This character of the stress–strain behavior presumably arises from the realization of a tendency for chain orientation and fibrillation of the materials during tensile deformation. An enhanced increase in the stiffness along with the rise of deformation of BC-based composites compared to the BC gel-films (see the differences between E_25–30%_ and E_10–15%_; Table 4) can be associated with the formation of a polyelectrolyte complex.

From a quantitative viewpoint, the mechanical characteristics of the gel-films can be divided into two groups. The films of wrung-out BC and of composites containing HA or ALG are low-stiffness materials with moduli up to 1.5 MPa and the ultimate stress tolerance of 0.4–1.6 MPa. The sample failure takes place at an extension of 50–60% (Table 4).

The films of the original BC and BC–CAR–CS differ dramatically from these other materials. Note, that the thicknesses of these two films are 10–15 times thinner than those of the other three materials (1.35–2.10 mm). This fact can denote a depressed swelling of these gel-films. Both the modulus and the ultimate stress values of these two films are more than two orders beyond those of the other three gel-films (Table 4) and accompany the depressed values of the ultimate deformation. The described differences in the mechanical properties of the two groups of gel-films presumably are exhaustively determined by the different extents of their swelling. Thus, the deformation and strength properties of the obtained composites do not fundamentally differ from the properties of the samples with a similar degree of swelling in water.

### 3.4. X-ray Diffraction Structure

**Figure 2:**
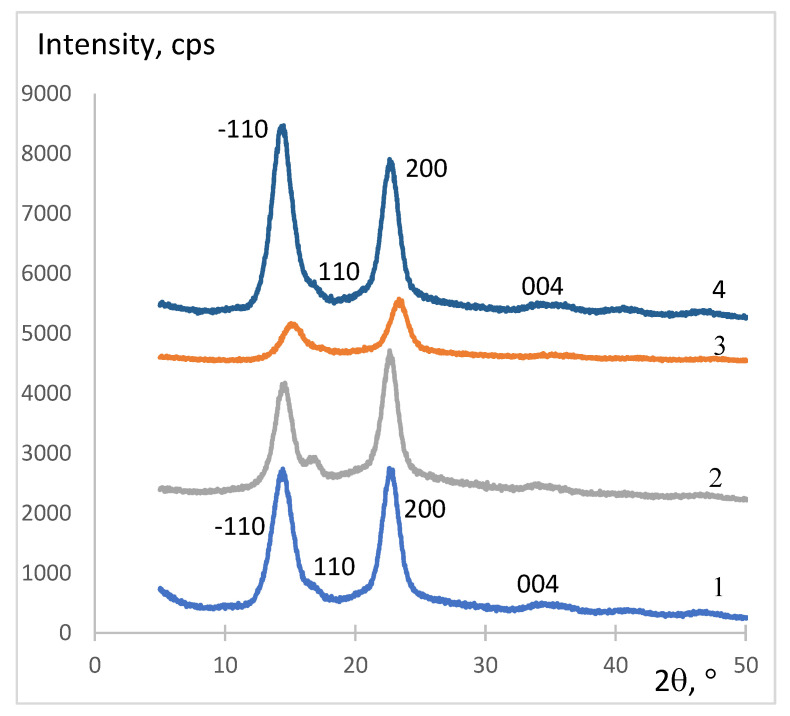
X-ray diffractograms of the bacterial cellulose composites: 1, BC; 2, BC–HA–CS; 3, BC–ALG–CS; 4, BC–CAR–CS.

X-ray diffraction analysis of the original BC sample and its composites showed that the structure of BC undergoes some changes during composite formation. The structural organization of composites depends on the type of anionic polysaccharide used (steric availability and amount and strength of ionic groups). The difference in the intermolecular forces in the BC composites affects the packing of the samples, which in turn affects the intensity of reflections in the X-ray diffraction patterns. Diffractograms of BC–HA–CS (Figure 2(2)) and BC–ALG–CS (Figure 2(3)) contain reflections typical of the original BC (2*θ* = 15° and 24°) (Figure 2(1)), but they are wider and less intense. The more intense reflections in the same areas found for BC–CAR–CS (Figure 2(4)) are indicative of stronger intermolecular interactions between the BC fibers due to their reinforcement by the PEC, which can be associated with the presence of stronger acidic -OSO_3_H groups in CAR compared to carboxyl groups in ALG and HA. An increase in the intensity of the reflection at 2θ = 15° for BC–CAR–CS (Figure 2(4)) indicates the prevailing orientation of BC nanofibrils along the (−110) crystallographic plane [34].

### 3.5. SEM Morphology of Composites

**Figure 3:**
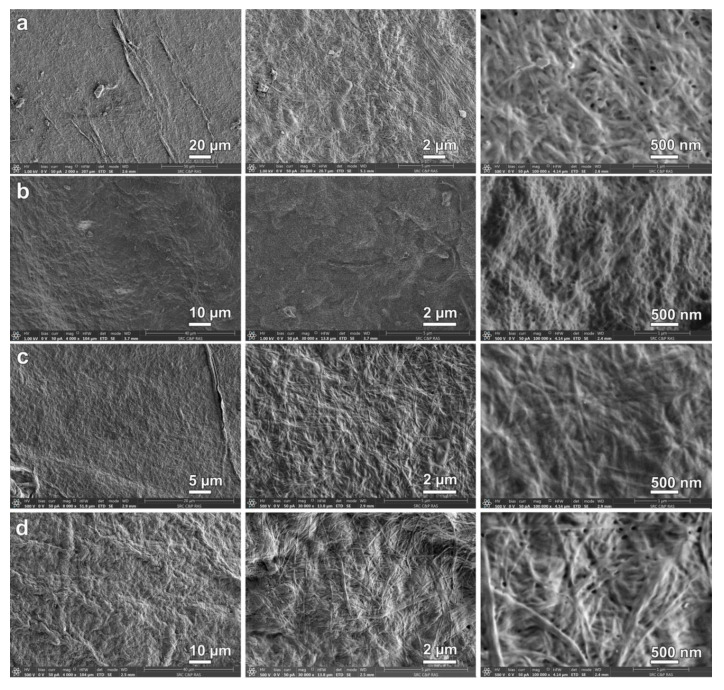
SEM images of surface of bacterial cellulose composites: BC (**a**); BC–ALG–CS (**b**); BC–HA–CS (**c**); and BC–CAR–CS (**d**).

Surface analysis of BC by SEM showed a random distribution of the fibers and of pores up to 100 nm in diameter, while cross-sectional images indicated ordered layers of fiber clusters [35]. The SEM surface images of the BC composites are shown in Figure 3. The morphology of BC–ALG–CS is a homogeneous network of nanofibers with evenly distributed pores of nanometer size. BC–HA–CS has a uniform surface morphology, and high magnification images clearly demonstrate nanofibers with almost completely filled interfibrillar spaces. Due to its structure and its interaction with BC, HA easily penetrates into the BC surface pores [36]. Significant changes were observed in the surface morphology in the case of BC–CAR–CS. Its textured and inhomogeneous surface morphology may indicate the formation of a more rigid PEC network.

**Figure 4:**
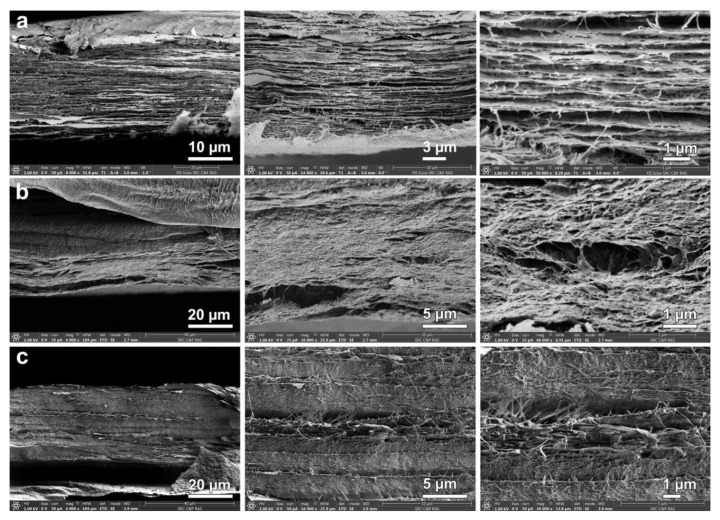
SEM images of cross-sections of: BC (**a**); BC–CAR–CS (**b**); and BC–HA–CS (**c**).

Analysis of the cleavage of BC and the BC composites (Figure 4) revealed significant changes in the morphology of the composites compared to the initial BC. Specifically, the boundaries between the layers disappear and the morphology becomes homogeneous, indicating a uniform penetration of polysaccharides into the BC.

### 3.6. Cytocompatibility Testing

Qualitative analysis of the cytocompatibility of scaffolds with cellular cultures revealed the following types of cells on the scaffold surfaces: adhered cells (isolated, multiple), colonies formed by these cells (monolayers, isolated spheroids, multiple spheroids), and conditionally non-adhered cells (isolated, multiple) that had presumably undergone apoptosis (Table 5). The average size of the spheroids was calculated by the maximum longitudinal size of at least 20 spheroid colonies for each sample (Table 6).

**Figure 5:**
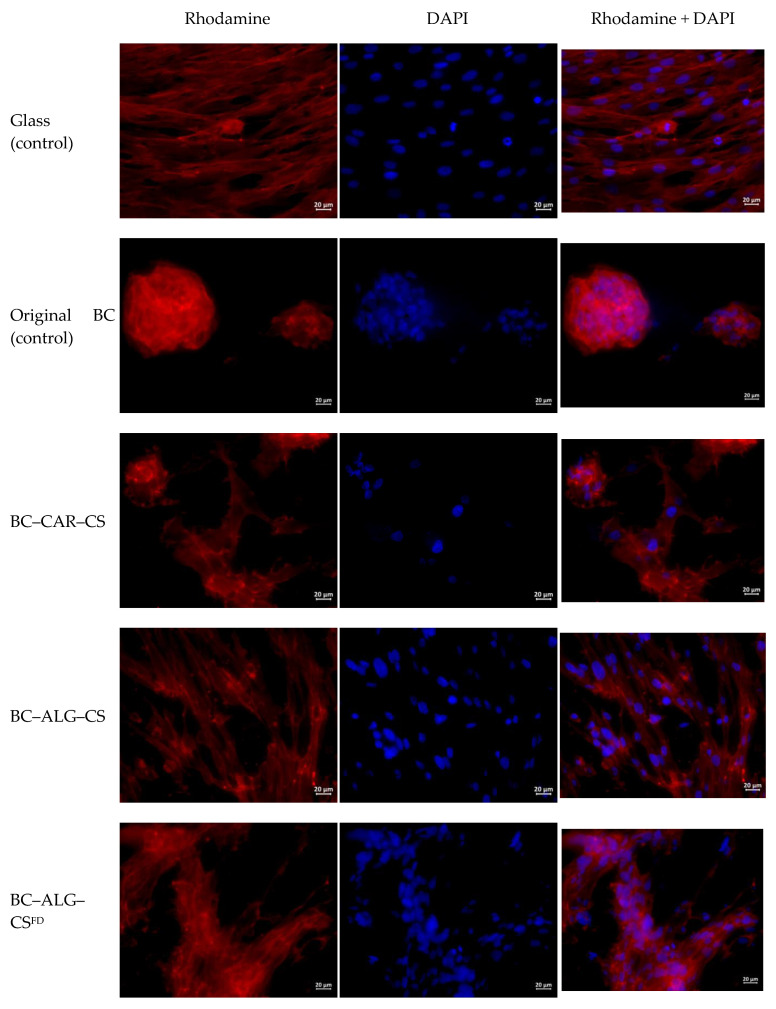

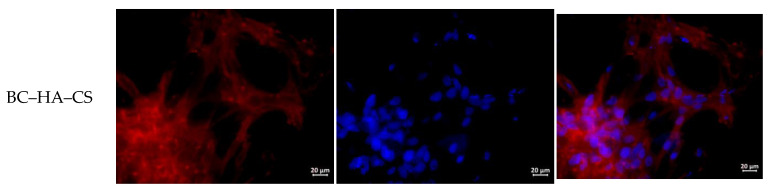
Multipotent mesenchymal stem cells adhered to the surfaces of glass and scaffolds. The fibrillar actin of the cytoskeleton was stained with rhodamine fluorochrome; nuclei were stained with DAPI. Combined two-channel image, magnification ×40.

**Figure 6:**
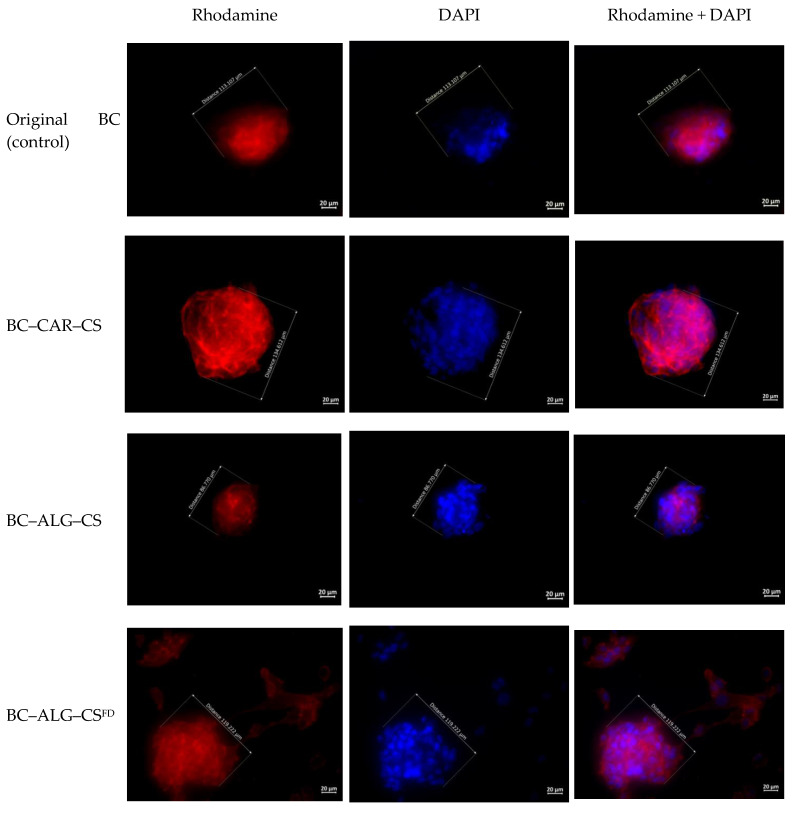

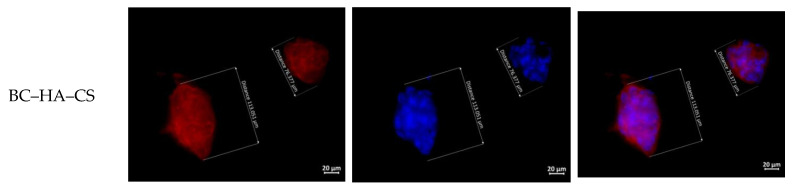
Multipotent mesenchymal stem cell spheroids formed on different scaffolds at three days after seeding (µm). The fibrillar actin of the cytoskeleton was stained with rhodamine fluorochrome; nuclei were stained with DAPI. Combined two-channel image, magnification ×40.

The cells on cover glasses (the reference group) spread out and grew on the glass surface to form a confluent/subconfluent monolayer (Figure 5 and Figure 6). These cells had a typical elongated shape with multiple appendages, and many cells were undergoing cell division. Cells in the BC-based scaffolds, by contrast, were located on the surface as individual isolated cells or as spheroid colonies of different sizes and containing multiple cells. The isolated cells had circular shapes with small peripheral protrusions. The cells located at the periphery of the spheroids had a spindle-like shape and few appendages. Single instances of spheroids connected via “bridges” of migrating cells were also observed.

The surfaces of the BC–CAR–CS scaffold were adhered with individual isolated cells, as well as multiple cells in the form of spheroids or small multilayered groups (Figure 5 and Figure 6). The majority of the single cells had a typical elongated shape with numerous appendages and areas of focal adhesion. A small portion of the single cells and the cells forming the small flat colonies had a circular shape with small peripheral protrusions. The cells located at the periphery of the spheroids had a spindle-like shape and many appendages. Single instances of the pairwise merging of spheroids were registered.

In the case of the BC–ALG–CS scaffold, the cells were located on the surface, mainly in the form of a monolayer with a confluence of 50–75% in certain areas or in the form of single spheroids (Figure 5 and Figure 6). The cells had a typical elongated shape with numerous appendages and areas of focal adhesion. Single cells had circular shapes with small peripheral protrusions. The cells located at the periphery of the spheroids had a spindle-like shape and many appendages, but these then gave way to a monolayer. No merging of the spheroids was detected.

In the case of BC–ALG–CS^FD^, the cells formed a monolayer on the surface; the colonies were predominantly flat with few spheroids (Figure 5 and Figure 6). The cells had a typical elongated shape with numerous appendages. Single cells had circular shapes with small peripheral protrusions. The cells located at the periphery of the spheroids had a spindle-like shape and many appendages. Many spheroidal colonies were connected via “bridges” of cells migrating from the spheroids.

In the case of BC–HA–CS, the cells formed a monolayer on the surface and predominantly flat colonies with few spheroids (Figure 5 and Figure 6). The cells had a typical elongated shape with numerous appendages. Many single cells had circular shapes with small peripheral protrusions. The cells located at the periphery of spheroids had a spindle-like shape and many appendages. Many spheroidal colonies were connected via “bridges” of cells migrating from spheroids. Single instances of a pairwise merging of spheroids were registered.

## 4. Discussion

Taking into account that MSCs are adherent cells, cell adhesion is the most important parameter in cell–material interactions, and adhesion convincingly characterizes cell functioning. We demonstrated the active phase of cell adhesion with cytoskeleton organization and the formation of focal adhesions between the cells and the materials. The mechanisms of this interaction have described been previously [37].

One unusual feature of MMSC cultures grown on the surface of CS scaffolds is the formation of spheroid colonies [38]. Spheroid colony formation is also typical of BC scaffolds [26]. This organization of cell colonies (in comparison with 2D monolayer culture) has a number of peculiarities concerning both the process of formation of the multicellular spheroid aggregates and the properties of the cells that constitute these spheroids.

In addition to spheroids found on the cover glasses, we also found many spheroids present on the surface of the modified polymers. MSC spheroids have previously been shown to have improved survival when compared to single cell suspensions [39], but they have less of a survival advantage than MSCs when grown in regular 2D cultivation conditions in vitro [40]. Granted, all unadhered MSCs are involved in apoptosis that leads to cell death. Taken together, our observations demonstrate (Table 5) that cell viability on the modified BC surface is worse than on control cover glasses but better than on unmodified BC.

The process of formation of spheroids on a CS surface differs from spheroid formation from cell suspensions or on a non-adhesive polymeric surface. The MMSCs first adhered to the CS scaffolds and spread along the surface using the numerous formed pseudopodia. The pseudopodia were then retracted and the cells formed multilayered spheroids [41,42] in an analogous manner to the spheroid formation observed on a polymer surface within several hours after initiating co-cultivation [41]. Spindle-like cells were then formed at the periphery of the spheroids and migrated along the adhesive surface of the CS scaffolds. The CS–HA scaffolds showed more pronounced cell migration than the other scaffold types. Previous studies have shown that no migration occurs on the surface of non-adhesive polymeric surfaces [43,44].

Cadherin-mediated homophilic intercellular interactions play an important role in the formation of multicellular spheroid aggregates [45,46]. The cells in spheroids differed from the MMSCs grown in 2D monolayer culture in terms of their phenotype and gene expression profiles. Cells in spheroid colonies had lower dimensions and volumes; the cells found outside the colonies were elongated, while the cells located inside the colonies were circular [38,44]. The number of viable cells found in spheroid colonies was directly related to the sizes of the colonies, and an increase in spheroid size was associated with an increased number of apoptotic and necrotic cells [44]. This observation could reflect the slow metabolism in the “nucleus” of the spheroid, where the rates of diffusion of nutrients and oxygen and the extraction of waste products are slower than at the boundary zone of a cell colony [38,40,41,47,48]. The number of viable cells in the spheroids on the CS scaffolds decreased gradually with time, whereas the majority of cells on the surface of the CS–HA scaffolds remained viable [41].

Spheroid size is determined by the mobility of the constituting cells. Cells that can move rapidly along the surface of a material form larger and greater numbers of spheroids. Spheroids can also possibly enlarge at the expense of smaller spheroids, as observed in experiments with CS–HA scaffolds but not with CS scaffolds (where no merging of colonies occurred) [41]. In turn, cell mobility on a scaffold surface depends on the type (source) of MMSC and the adhesive characteristics of the polymer surface (these characteristics are determined by the composition of the scaffold). Thus, spheroids were larger when derived from MMSCs obtained from human fat tissue than from the placenta, and they were larger when grown on CS–HA scaffolds than on the pure CS samples. Increases in the percentage of HA in the composite led to further increases in the spheroid size [41].

The gene expression profile of MMSCs in spheroid colonies demonstrated an increased expression of genes associated with hypoxia, angiogenesis, inflammation, stress responses, and redox signaling [38]. These types of changes are thought to facilitate an increased capability for differentiation and more pronounced anti-inflammation, antifibrotic, regenerative, and reparative properties than is observed in MMSCs in two-dimensional culture [38,44,49]. At present, however, the molecular mechanisms determining these changes in gene expression are still poorly understood [38].

Spheroid MMSC colonies were observed on the surface of all the polymer scaffolds. The largest spheroids were found on the scaffolds of the reference group (BC), BC–CAR–CS, and BC–ALG–CS^DF^ (*p* < 0.05, Mann–Whitney test). Direct (without “bridges”) or mediated (through “bridges” of migrating cells) merging of spheroids was observed for all scaffolds. The sole exception was BC–ALG–CS, which showed singular spheroids localized on the surface at large distances from each other. Indirect signs of migration (spindle-like cells with peripheral appendages and “bridges” of cells between spheroids) were evident for cells from the spheroids in all cases, but these signs were more pronounced in the experiments with BC–ALG–CS^FD^, BC–ALG–CS, and BC–CAR–CS. Circular cells with small peripheral protrusions localized near other cells and colonies were registered on the surfaces of all scaffolds, and these cells had apparently undergone apoptosis (final stages). The largest numbers of these cells were seen on the surfaces of the control sample (BC) and BC–HA–CS.

The obtained data indicate that the properties of the matrix material depend on its surface morphology, porosity, and hydrophilicity. Cell compatibility was greater with the BC composite materials than with the original BC sample. The BC-based composites showed different propensities for supporting the growth of stem cells, and this difference was related to the anionic polysaccharide structure and its interaction with CS. Among the obtained composites, the best characteristics were found for BC–ALG–CS, which supported adherence of numerous MMSCs to its surface, predominantly in the form of monolayer colonies. (The best cytocompatibility was also seen with BC-based scaffolds impregnated with CS and ALG, presumably due to the increased swelling degrees and porosities of these composites.) The method used for drying of composites also influenced the cell growth, as some changes were observed between BC–ALG–CS^FD^, which was freeze-dried, and BC–ALG–CS, which was air dried at room temperature.

## 5. Conclusions

Bacterial cellulose was confirmed as a useful component for the preparation of composites with natural polysaccharides, as the composites form via intermolecular interactions without the need for crosslinking agents. Modification of BC by successive impregnation with solutions of oppositely charged anionic polysaccharide (ALG, HA, or CAR) and the cationic polysaccharide CS yields composites with differing morphologies, structures, and swelling and porosity characteristics. These differences are related to the properties of the PEC formed by electrostatic interactions between CS and polyanions in BC composites. The degree of crosslinking in a polyelectrolyte network depends on the complementarity of the two polyelectrolytes, the steric availability, and the number and strength of oppositely charged ionic groups. In the case of the PEC formed by CS and CAR containing sulfate groups, the degree of ionic crosslinking is much higher than that of the PEC formed from CS and polyanions (ALG and HA) that contain carboxylate groups. The difference in the properties between ALG and HA can be associated with the structural features of these anionic polysaccharides.

The BC-based composites demonstrate higher cytocompatibility than the original BC sample, with the best characteristics found for the BC–ALG–CS composite in this study. The choice of drying method for a composite sample can also influence the morphology of the cells cultivated on its surface, as well as the type of colonies formed by those cells.

## Figures and Tables

**Table 1:** Preparation and properties of bacterial cellulose composites. (For all samples): The final treatment of the samples was 2% ammonia in ethanol, washing with ethanol, and drying at room temperature.

**Sample**	**Treatment of BC**	**Swelling in Water, g/g**
original BC	-	1.9
BC–CAR–CS*	1. immersion into 1% solution of CAR (2 h) 2. immersion into 1% solution of CS (1 h)	1.5
BC–CAR–CS	1. immersion into 0.5% solution of CAR (2 h) 2. immersion into 1% solution of CS (1 h)	2.8
BC–HA–CS*	1. immersion into 3% solution of HA (2 h) 2. immersion into 1% solution of CS (1 h)	16.0
BC–HA–CS**	1. immersion into 0.5% solution of HA (3 days) 2. immersion into 1% solution of CS (1 h)	5.1
BC–HA–CS	1. immersion into 3% solution of HA (1 h) 2. immersion into 1% solution of CS (30 min)	14.1
BC–ALG–CS	1. immersion into 2% solution of ALG (2 h) 2. immersion into 1% solution of CS (30 min)	24.0

**Table 2:** Composition of bacterial cellulose samples.

Sample	BC, %	Anionic polysaccharide, %	CS, %
BC–ALG–CS	71	26	3
BC–HA–CS	62	34	4
BC–CAR–CS	72	20	8

**Table 3:** The porosity of bacterial cellulose composites.

Parameter	BC–HA–CS	BC–CAR–CS	BC–ALG–CS
Average logarithmic pore radius, nm	1.70	2.55	1.33
Average pore radius, nm	339	752	472
Porosity over weight, cm^3^/g	1.81	0.943	1.52
Porosity over volume, cm^3^/cm^3^	0.585	0.445	0.606
Meso- and macro-pore surface over weight, m^2^/g	445	91.9	392
Meso- and macro-pore surface over volume, m^2^/cm^3^	144	43.3	156
Total pore surface over weight, m^2^/g	951	91.9	1028
Total pore surface over volume, m^2^/cm^3^	308	43.3	409

**Table 4:** The mechanical properties of bacterial cellulose composites.

Sample	Thickness, mm	E_10–15%_, MPa	E_25–30%_, MPa	σ_b_, MPa	ε_b_, %
wrung-out BC	1.35	1.26 ± 0.24	1.47 ± 0.11	1.61 ± 0.25	54 ± 1
original BC	0.13	205 ± 23	315 ± 29	62 ± 3	32 ± 2
BC–HA–CS*	2.10	0.35 ± 0.06	0.69 ± 0.11	0.43 ± 0.07	52 ± 2
BC–ALG–CS	2.05	0.41 ± 0.07	1.17 ± 0.17	0.55 ± 0.04	61 ± 3
BC–CAR–CS	0.13	129 ± 12	192 ± 18	57 ± 4	37 ± 2

**Table 5:** Characteristics of multipotent mesenchymal stem cells and cell colonies formed on the surface of the scaffolds.

Sample	Non-Adhered Cells	Adhered Cells	Spheroids	Prevailing Type of Colony
Glass (control)	-	multiple	-	monolayer
Original BC (control)	multiple	multiple	multiple	spheroids
BC–CAR–CS	isolated	multiple	multiple	spheroids
BC–ALG–CS	isolated	multiple	isolated	monolayer
BC–ALG–CSFD	isolated	multiple	multiple	monolayer + spheroids
BC–HA–CS	multiple	multiple	multiple	monolayer + spheroids

**Table 6:** The average and maximal longitudinal size of multipotent mesenchymal stem cell spheroids formed on different scaffolds at three days after seeding, µm. Reliability of the changes in comparison with the reference group (Mann–Whitney): * *p* < 0.05.

Sample	Mean ± SD	Max Longitudinal Size of Spheroids, μm
Original BC (control)	117 ± 50	243
BC–CAR–CS	133 ± 48	216
BC–ALG–CS	81 ± 14 *	103
BC–ALG–CSFD	122 ± 64	301
BC–HA–CS	85 ± 20 *	123
